# Grand challenges in inorganic chemistry: toward better life quality and a more sustainable world

**DOI:** 10.3389/fchem.2013.00002

**Published:** 2013-03-11

**Authors:** Jean-Claude G. Bünzli

**Affiliations:** Laboratory of Lanthanides Supramolecular Chemistry, Institute of Chemical Sciences and Engineering, École Polytechnique Fédérale de LausanneLausanne, Switzerland

Initially limited to the study of minerals, salts, and oxides, inorganic chemistry (IC) emerged as a major actor in technology and sciences during the second part of the last century. Sulfuric and phosphoric acids, ammonia, lime, and hydraulic cement, for instance, are among the top-20 most manufactured chemicals. Electronic industry and solar energy conversion rely on various forms of highly pure silicon while more sophisticated compounds are vital to medical diagnosis, imaging and therapy, not to mention catalysts which are indispensable in industrial chemistry. On a more fundamental level, inorganic compounds intervene in biological processes and the knowledge of their structure and reactivity is crucial to the understanding of associated biochemical reactions (Rebilly and Reinaud, 2012). Highly sophisticated functional materials often contain metal ions and are assembled following the principles of metallosupramolecular chemistry (Ariga et al., 2012). These are only a few examples of the ubiquitous roles played by inorganic chemistry. Although IC has presently grown up to a high level of sophistication, there are still many scientific issues to be resolved and future perspectives and needs are highly challenging. In the following, we concentrate on three major aspects only.

## Life mechanisms and health issues

Many questions and challenges are being tackled in relationship with mechanisms and entities essential to life. Among relevant examples, elucidating the structure of metalloenzymes and its relationship with reactivity plays a central role. Furthermore, unraveling the interaction between metals and nucleic acids, particularly DNA and RNA, is a key issue when it comes to anticancer drugs. The archetype cis-platin drug continues to generate a wealth of studies, but starts to face competition from protein and enzyme-inhibitor gold and ruthenium complexes (Che and Siu, 2010). Another example is the deciphering of metal ion trafficking and homeostasis (Mbatia and Burdette, 2012) in living cells since this has direct relationship with how cells manage the uptake, distribution, and storage of metal ions while avoiding toxicity (Lutsenko, 2010). As a follow up, trying to understand the concentration/benefit/toxicity relationship of metal ions which are essential nutrients is becoming an urgent field of research. Conversely, if the concentrations of toxic elements such as cadmium, mercury, or lead in human blood are well-known for the average population, their connection with the etiology of grievous human diseases, such as Alzheimer's and Parkinson's diseases, is not well-understood and getting more precise information on this link may be one of the greatest post-genomic challenges for bioinorganic chemistry (Gailer, 2012).

Diagnosis and treatment of cancer and other diseases are presently focusing on individualized approaches which, in turn, necessitate better and faster pathological analyses as well as highly contrasted, real-time bioimages. Non-invasive methodologies are required for perturbing the investigated organs as little as possible and both magnetic resonance imaging (MRI) and optical emissive probes are proven outstanding tools. Metal complexes as radiopharmaceuticals for positron emission tomography also generate useful images of tumors (Huang and Davis, 2011). Contrast agents for MRI featuring gadolinium complexes are commonplace in medical diagnosis since the mid-1980s and despite large improvements in instrumentation resulting in much higher detection sensitivity, they are still needed for both general purposes (Boros et al., 2012) and targeting specific organs or receptors (Hahn et al., 2011), as well as for monitoring drug delivery (Caravan, 2009). However, MRI has shortcomings and the main idea is to develop integrated multimodal techniques, for instance relying on both MRI and optical (luminescent) probes (Ai, 2011) or for theranostics purposes (Janczewski et al., 2011). In this respect, nanoparticles are becoming privileged vectors (Kelkar and Reineke, 2011).

A complementary aspect is bioanalysis and cell imaging, the needs for which are growing exponentially. Time-resolved spectroscopy and microscopy are ideal tools to suppress autofluorescence and organic stains are often being replaced by transition metal (Rosch and Baum, 2011) and lanthanide (Bünzli, 2010; Lo et al., 2012; Neugebauer et al., 2012) complexes. The main challenge here is to design robust complexes which dissociate as little as possible under biological conditions and feature bioconjugation capabilities in order to specifically target an analyte or a biomarker. Nanoparticles, again, will take up an increasingly prominent role in this field (Gnanasammandhan and Zhang, 2012; Hotzer et al., 2012).

## Functional materials

This subject covers widely different themes, from self-repairing concrete (Mihashi and Nishiwaki, 2012) to counterfeiting tags (Lindsey et al., 2011) and has strong links with nanotechnology. One recurrent aspect though is catalysis, particularly homogeneous catalysis which still has to acquire the robustness of heterogeneous systems. To this end, active molecules are often grafted onto solids such as zeolites or silica (Gross et al., 2012); chiral catalysts are particularly sought for and chiral anions are well-suited for this purpose since they act through either simple electrostatic or hydrogen-bonding interactions (Phipps et al., 2012). Another facet is represented by porous materials, self-assembled from metal ions and suitable organic ligands which are in high demand with respect to gas storage, particularly carbon dioxide, methane, or hydrogen, all related to environmental and energy issues (Almeida Paz et al., 2012).

Inorganic molecular systems with specific structures are the core of many functional materials, especially when electronic or lighting devices are concerned. Metallomesogens which combine the properties of metals (luminescence, magnetism) with those of liquid crystals (fluidity, anisotropy, response to electric fields) belong to a class of compounds which may yield interesting features, particularly with respect to magnetic switching and magneto-optical data storage (Binnemans, 2013). Another fascinating development is the design of single molecule magnets (SMMs) the properties of which can be modulated thanks to weak interactions in multidimensional supramolecular arrangements, e.g., through hydrogen bonds. These materials are foreseen for information storage as well as for molecular electronics and photonics. Much knowledge has been gathered during the past two decades on SMMs, but many improvements are still needed before these materials can reach practical applications (Jeon and Clerac, 2012; Luzon and Sessoli, 2012). Luminescence is detectable with high sensitivity so that many analytical processes and sensors rely on it, as well as molecular logics where computing concepts are embedded into molecular structures (De Silva and Uchiyama, 2011). Here too, laboratory systems have to be perfected before they reach practicability. Safety and traceability aspects are becoming unavoidable issues and upconversion nanoparticles make up an increasing share of the related tags and codes which are needed for these purposes, for instance in security inks (Meruga et al., 2012). Another aspect of luminescent materials is the search for multicolor long-persistence phosphors for watch dials, safety signs, and lighting, or even for bioimaging (Wang et al., 2013). Finally, surface protection and derivatization of all kind of materials also draw hefty attention, not only with respect to preventing corrosion but, also, to getting self-cleaning photoactive surfaces (windows, pavements) based on semi-conductors such as titanium oxide (Mills et al., 2012).

## Energy-related topics

A major challenge faced by humanity is to find sustainable and abundant energy resources. Solar energy represents a plentiful, seemingly cheap, source of energy and since the 1950s, light-to-energy conversion is a major research theme of inorganic chemistry. Silicon or other semi-conductors have been designed to achieve the largest possible conversion efficiencies, up to 44% presently for multijunction cells under concentrated irradiation (Green et al., 2012). More recently, dye-sensitized solar cells with mesoporous titanium dioxide as photoanode and a transition metal complex as sensitizer have attracted hefty interest because they perform better under diffuse light and are more flexible, which allows one to affix them on windows. Considerable efforts are still needed to boost the conversion yield and the durability of these photovoltaic devices, either intrinsically, or by means of wavelength converting materials; this in turn implies considerable efforts related to solid state and coordination chemistry (Cates et al., 2012), as well as to materials design (Robson et al., 2012). The main challenge though is not to reach the highest possible conversion yield but, rather, to achieve “grid parity,” that is a price per watt equal or lower than the price of electricity generated by other means, approximately 1 US $/W. In addition, photostability should be given high priority too.

Energy-saving lighting is an additional challenge for inorganic chemists since it relies on efficient photostable phosphors made up of doped inorganic oxides or salts, the composition of which has to be optimized, which means exploring millions of potential chemical formulae (Nazarov and Noh, 2011).

Photocatalysis is another facet of the problem (Fujishima et al., 2008) with higher ambition: in addition to producing solar fuels (typically hydrogen), one gains storage capability, a real Holy Grain since sunlight is not always available. Main efforts are focusing on water splitting (Walter et al., 2010; Valdes et al., 2012), an ideal process since energy production from hydrogen and oxygen only generates innocuous water as waste. Transition metal chemists have dealt with the problem for more than 40 years, when TiO_2_ was reported to help electrochemical photolysis of water, and conversion yields start to be sizeable, albeit many difficulties remain to be addressed. In particular, multijunction configurations, which perform best, require carefully engineered contacts. The size and shape of the micro- and nano-structures used in these devices is of paramount importance and need full optimization. The search for new and better performing photocatalysts also has to be added to the “to-do” list. Especially (and this is also valid for photovoltaics) because of foreseeable shortcomings in rare elements which are often the core components of these devices, earth-abundant materials should be developed without losing too much performance, a real conundrum! Finally the throbbing aspect of cost should also be kept in mind while developing “artificial leaves” (Nocera, 2012) or photocatalysts for water and air depollution, for which plasmonics may help (Wang et al., 2012).

## Conspectus

The three aspects presented above have in common the need for new and better performing molecules and materials. This necessitates attention to be given to synthetic methods. Techniques such as atom transfer radical polymerization for producing functional polymers, chemical vapor deposition, ink-jet printing, or layer-by-layer assembly for derivatizing surfaces or fabricating multifunctional films, block-copolymer templating of inorganic materials for obtaining nanoporous and mesoporous materials, sol-gel and solvothermal processes for growing nanoparticles with controlled size and properties, as well as self-assembly processes, to name but a few, will all need to be perfected in order to meet the stringent future needs of inorganic chemistry.
